# Application of synthetic lipid droplets in metabolic diseases

**DOI:** 10.1002/ctm2.1441

**Published:** 2023-11-23

**Authors:** Pengxiang Zhao, Zichen Zhao, Ziwei Yu, Lupeng Chen, Yi Jin, Jian Wu, Zhuqing Ren

**Affiliations:** ^1^ Key Laboratory of Agriculture Animal Genetics Breeding and Reproduction of the Ministry of Education, College of Animal Science Huazhong Agricultural University Wuhan Hubei P. R. China; ^2^ College of Animal Science and Technology Shandong Agricultural University Taian Shandong P. R. China; ^3^ Frontiers Science Center for Animal Breeding and Sustainable Production Wuhan Hubei P. R. China; ^4^ Hubei Hongshan Laboratory Wuhan Hubei P. R. China

**Keywords:** artificial lipid droplets, lipid droplets, metabolism diseases, synthetic biology

## Abstract

**Background:**

The study and synthesis of membrane organelles are becoming increasingly important, not only as simplified cellular models for corresponding molecular and metabolic studies but also for applications in synthetic biology of artificial cells and drug delivery vehicles. Lipid droplets (LDs) are central organelles in cellular lipid metabolism and are involved in almost all metabolic processes. Multiple studies have also demonstrated a high correlation between LDs and metabolic diseases. During these processes, LDs reveal a highly dynamic character, with their lipid fraction, protein composition and subcellular localisation constantly changing in response to metabolic demands. However, the molecular mechanisms underlying these functions have not been fully understood due to the limitations of cell biology approaches. Fortunately, developments in synthetic biology have provided a huge breakthrough for metabolism research, and methods for in vitro synthesis of LDs have been successfully established, with great advances in protein binding, lipid function, membrane dynamics and enzymatic reactions.

**Aims and methods:**

In this review, we provide a comprehensive overview of the assembly and function of endogenous LDs, from the generation of lipid molecules to how they are assembled into LDs in the endoplasmic reticulum. In particular, we highlight two major classes of synthetic LD models for fabrication techniques and their recent advances in biology and explore their roles and challenges in achieving real applications of artificial LDs in the future.

## Graphical abstract

1. Lipid droplets are central organelles in lipid metabolism.

2. Lipid droplets can be investigated by both in vivo labelling and in vitro reconstitution methods.

3. Function of lipid droplets changes dynamically with cellular nutritional status.

4. Synthetic lipid droplets are emerging as a new breakthrough in metabolic research.

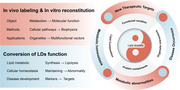

## 1 BACKGROUND

The membrane system is critical to cellular function, not only serving as a barrier between the cell and the environment but also providing support for its metabolism and molecular interactions. Intracellular or extracellular signalling is perpetuated through the interaction of certain molecules with specific membrane regions or transmembrane receptors. Thus, the membrane system is the basis of cellular physiology. However, under different cellular nutrient states, membrane systems have different lipid compositions and protein components and diverge in the abundance of subcellular compartments. This dynamic character creates a great challenge for the research of biological membranes and membrane proteins.

The large majority of organelles are composed of phospholipid (PL) bilayers, but lipid droplets (LDs) are an exception. LDs are highly conserved organelles between species, from bacteria to mammals, and consist of a neutral lipid (NL) core and a monolayer of PLs loaded with a variety of proteins on its surface. In general, the NLs are synthesised by cells in response to nutrient excess or various stresses and are subsequently packaged into LDs with PLs and proteins in the endoplasmic reticulum (ER).^1^ Thus, LDs are present in a variety of cell types and play a critical role in multiple cellular processes, including lipid and protein turnover, membrane trafficking and ER stress. Additionally, LDs are associated with a range of common diseases, such as cancer, obesity, nonalcoholic fatty liver disease (NAFLD) and cardiovascular disease.^2^ While the production of NLs, PLs and proteins has been extensively studied, the mechanisms underlying LD formation, including the initiation of LD generation, the generation process and the mode of transport, remain incompletely understood.^3^ However, it is now well established that LDs are highly dynamic organelles that originate from the ER and that their size, lipid and protein composition, and functions can be modified in response to cellular nutritional status and through interactions with other organelles.^4^ Like other organelles, LDs require intracellular labelling and tracing to be studied effectively.

In addition to conventional fluorescent protein labelling and chemical probes, synthetic biology has also been applied to the research of LDs. In vitro remodelling of LDs has emerged as a promising new approach for exploring LD function. Synthetic LDs are excellent membrane mimetics that can qualitatively or quantitatively reflect the adaptive changes of LDs to different cellular nutritional states by adding specific NL and PL components.^5^ The in vitro synthesis and assembly of this subcellular organelle explained the metabolic pathways of lipids and illuminated the impact of the latter on the cell membrane system in disease. More importantly, this study lays the foundation for the generation of more complex bio‐engineered synthetic cells in the future. In this review, we provide a comprehensive overview of the assembly and function of endogenous LDs, from the generation of lipid molecules to how they are assembled into LDs in the ER. In particular, we highlight two major classes of synthetic LD models for fabrication techniques and their recent advances in biology, and explore their roles and challenges in achieving real applications of artificial LDs in the future.

## 2 COMPOSITION OF ENDOGENOUS LIPID DROPLETS

### 2.1 Synthesis of neutral lipids

**FIGURE 1:**
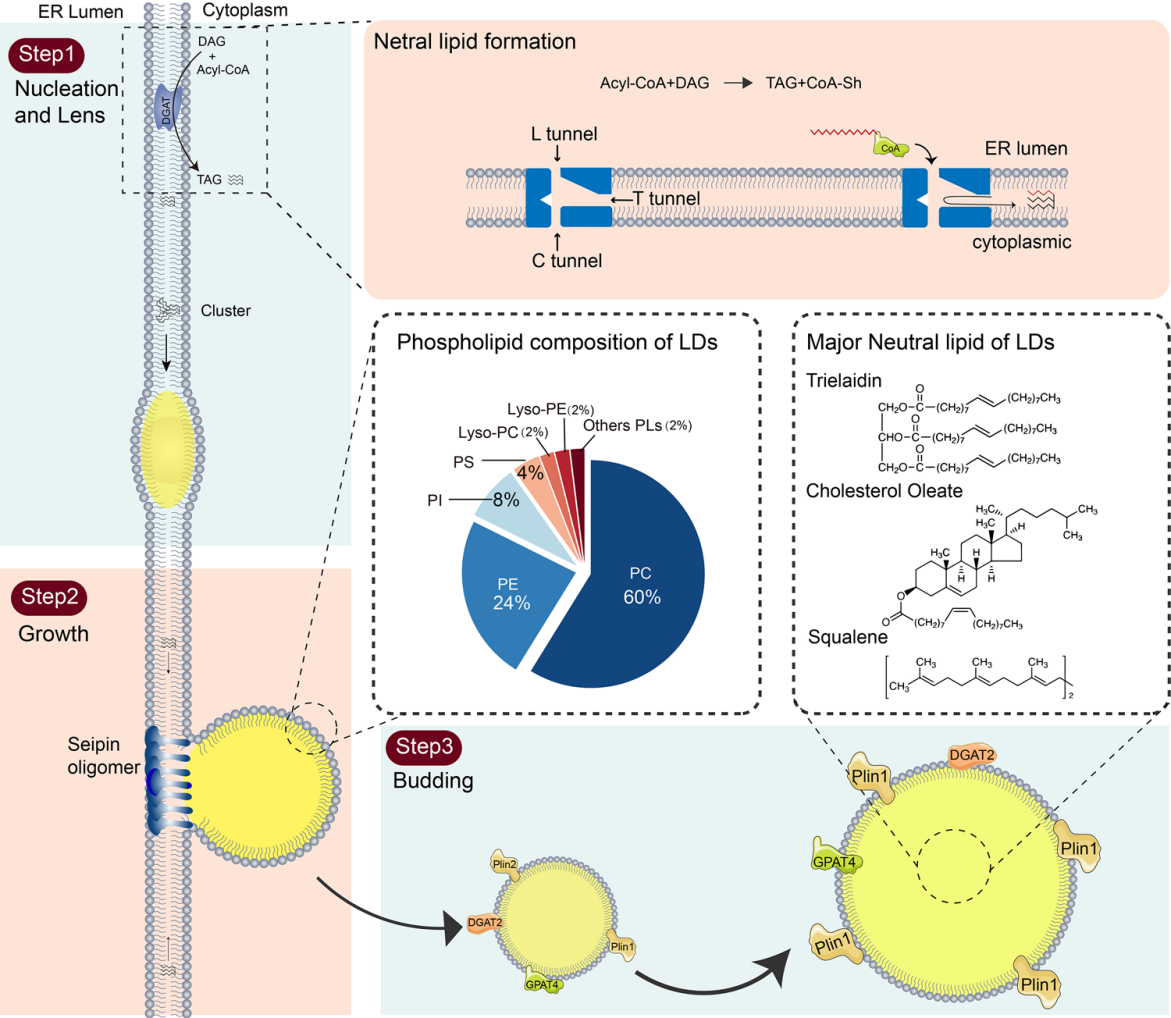
Lipid droplet (LD) production and lipid composition. There are three main steps in LD generation. The first step is nucleation and lens. In this process, neutral lipids (NLs) are synthesised in the endoplasmic reticulum by diacylglycerol acyltransferase (DGAT). T and C channels can supply the entrance for acyl‐CoA. The reaction is performed at the intersection of the two tunnels with the active residue His460. The export of the products remains to be investigated and it is currently found that CoA‐SH is released to the cell membrane through the C channel, while diacylglycerol (DAG) can reach the membrane or the lumen through the T and L channels, respectively. Second step is growth. NLs accumulate continuously in the lens and bud out to the cytoplasmic side. This process is mainly regulated by seipin protein and phospholipids (PLs). Third step is budding, in which LDs bud from the endoplasmic reticulum (ER) and become large in the cytoplasm by DGAT2.

LD formation in eukaryotes and some prokaryotes typically begins with the synthesis of NLs within the ER bilayer. However, in plants, NL synthesis may occur within the inner envelope of plastids, which is similar to the plasma membrane of prokaryotes.^6^ Triacylglycerols (TAGs) are the most abundant NLs in eukaryotes, followed by sterol esters (SEs). The primary synthetic enzymes for these NLs are diacylglycerol acyltransferase 1/2. They share similarities in their catalytic mechanisms and contain a bilayer intramembrane catalytic compartment with three entrances: the cytoplasmic (C tunnel), the ER lumen (L tunnel) and the transmembrane (T tunnel).^7^ Although the mode of catalysis is not fully understood, acyl‐CoA enters through the C channel, while diacylglycerol (DAG) l may enter through the L or T channel. The final reaction product (TAG) exits through the T channel within the bilayer.^8^ After synthesis, NLs integrate with PLs and diffuse within the ER bilayer, providing the fluidity required for subsequent LD formation (Figure 1).^9^

### 2.2 Phospholipid composition

The surface of LDs is composed of a monolayer of PLs and proteins, comprising over 100 different types of PLs and several specific LD proteins. This surface serves as a protective boundary between the hydrophilic cytoplasmic environment and the NLs contained within the LDs, while also influencing their overall function. The composition of LD PL monolayers is heterogeneous, and it depends on the nutrient status of the cell and the expression of PL metabolising enzymes.^10^ In general, phosphatidylcholine (PC) is the most abundant, constituting approximately 60% of the total content.^11^ Phosphatidylethanolamine (PE) and phosphatidylinositol (PI) are the next most abundant components, at 24% and 8%, respectively, while phosphatidylserine and lysolecithin (lyso‐PC or PE) each account for 4%.^12^ While other PLs are generally less enriched in LDs, some studies have reported a high concentration of sphingomyelin in LDs in fibroblasts^13^ and adipocytes, suggesting that the PL composition of LDs may vary across different tissues. DAG can accumulate abundantly in LDs, while its upstream precursor, phosphatidic acid (PA), is only transiently detected. These observations are consistent with the function of LDs as important transient intermediates in lipid biosynthesis.

PLs are responsible for creating a separation between the aqueous environment of the cytoplasm and the hydrophobic core of LDs. This is achieved by orienting the hydrophilic head group towards the outer cytoplasm and the hydrophobic tail towards the inner side. LDs maintain a spherical shape in most cell types, but they may aggregate into grape‐like structures through the cytoskeletal system.^14^ Thus, the encapsulation of LDs by a PL monolayer is crucial for maintaining organelle integrity and preventing the spontaneous fusion of LDs with other membrane‐bound organelles. Inhibition of PC synthesis leads to an increase in TAG synthesis and LD expansion.^15^ This is due to the activation of the SREBP‐1 pathway, which increases lipid synthesis when PC levels in the ER are depleted.^16^

PL monolayers on the surfaces of LDs not only serve as a boundary, but also play a crucial role in recruiting proteins to LDs surfaces. Multi‐omics analysis conducted in yeast has revealed that both nutrient status and genetics influence the components of LDs.^17^ Adipogenesis and lipolysis both result in a significant turnover of DAG: adipogenesis involves 1,2‐DAG, lipogenesis produces mainly 1,2‐DAG and lipolysis generates 1,3‐DAG or 2,3‐DAG.^18^ These distinct DAGs can serve as different recruitment signals for the proteins. It has been demonstrated that the presence of DAG in LDs and ER is critical for the recruitment of perilipin proteins (perilipin 3, 4, 5).^19^ The direct interaction of Plin2 with PC, sphingolipids and cholesterol may facilitate its localisation to LDs.^13^, ^20^ The recruitment of Plins proteins is also affected by the mobility of the surface monolayer. In addition to the membrane lipid composition, the type of NLs also affects the recruitment of proteins, which depends on the protein's recognition of core lipids. This recognition is supported by the lower packing density of PL monolayers in LDs compared to other intracellular membrane systems.^21^

## 3 LIPID DROPLETS AND METABOLISM DISEASES

LDs sense fluctuations in nutritional status through the metabolic turnover of cellular lipids. Therefore, various metabolic states in different tissues result in the existence of distinct subpopulations of LDs that vary in size, number, subcellular localisation and composition.^22^ During the fasted state, TAG are hydrolysed to free FAs by neutral lipolytic enzymes (i.e., lipolysis) and/or lysosomal lipases (i.e., lipophagy) located on the surface of LDs.^23^ In particular, Plin2‐mediated LD‐lysosome selective autophagy (i.e., lipophagy) plays a critical role in triglyceride mobilisation during starvation.^24^, ^25^ Conversely, LD generation is stimulated to store lipids and alleviate lipotoxicity in response to nutrient overload or cellular stress.^26^ Notably, excessive neuronal activation in the brain has been associated with fat and lipid peroxide formation. To protect neuronal membranes, the resulting lipid peroxide is transferred to LDs in astrocytes.^27^, ^28^

**FIGURE 2:**
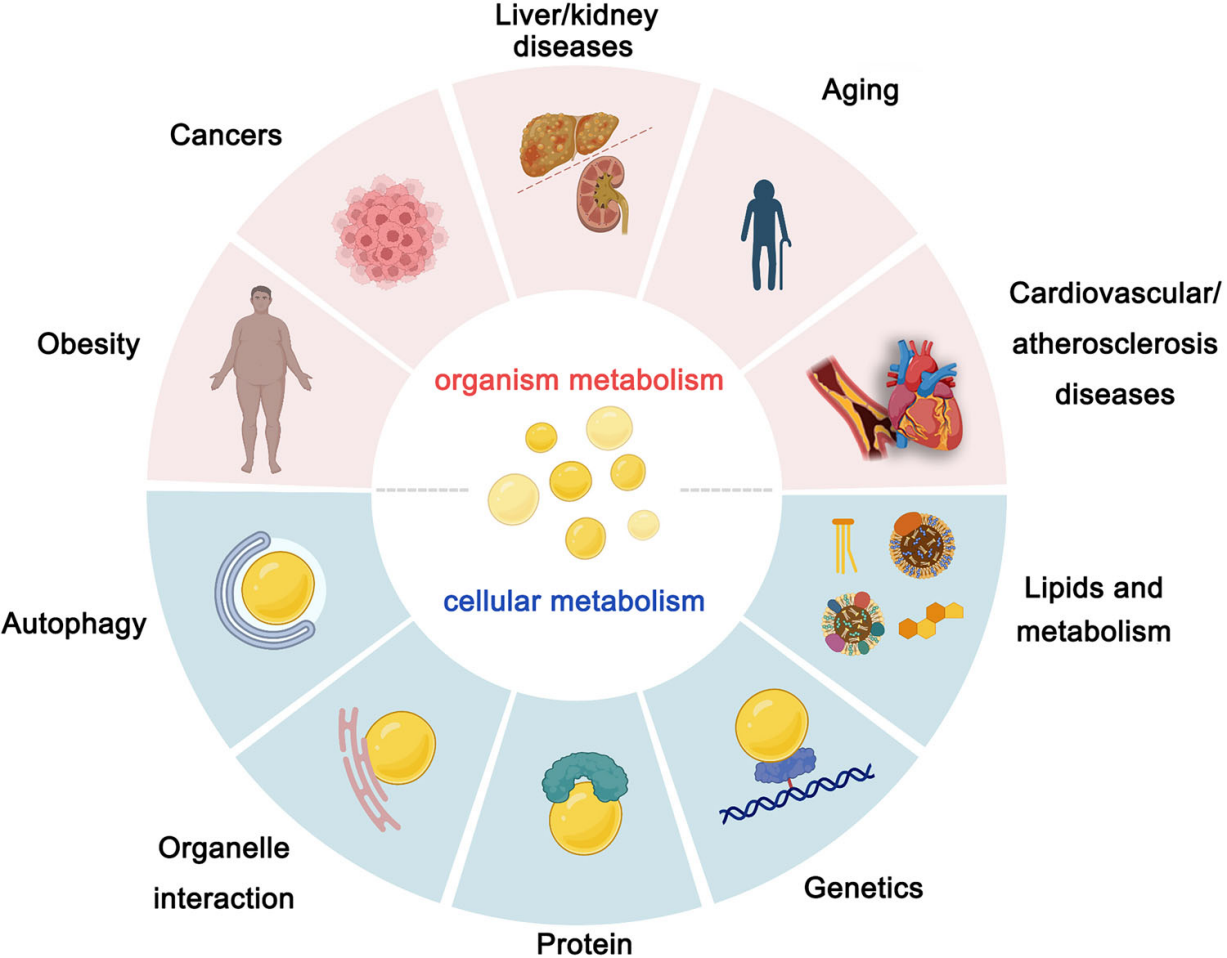
The function of lipid droplets (LDs). Based on the increasing number of findings on LDs emerging from several different disease models, we propose to associate LDs with several disease features. Although the role of LDs in these events needs to be studied in more depth, these associations may be used to explore future therapeutic approaches.

LDs perform various functions by interacting with other organelles, such as the ER, mitochondria and peroxisomes. Of particular note are the dynamic connections between LDs and the ER, which have a profound impact on cellular function. Mutations in genes related to ER function lead to the accumulation of LDs,^29^ and LDs offer protection to cells during ER stress. This includes the removal of misfolded or damaged proteins, the buffering of lipid toxicity, the restoration of ER lipid homeostasis, and the regulation of autophagic processes.^30^ LDs are also present in the nucleus and are involved in regulating PC synthesis, which is critical for maintaining the balance of membrane transport and ER pressure.^31^ Furthermore, LDs can protect Drosophila embryos from DNA damage by transferring excess histones, indicating that LDs may play a role in cell proliferation (Figure 2).^32^

**FIGURE 3:**
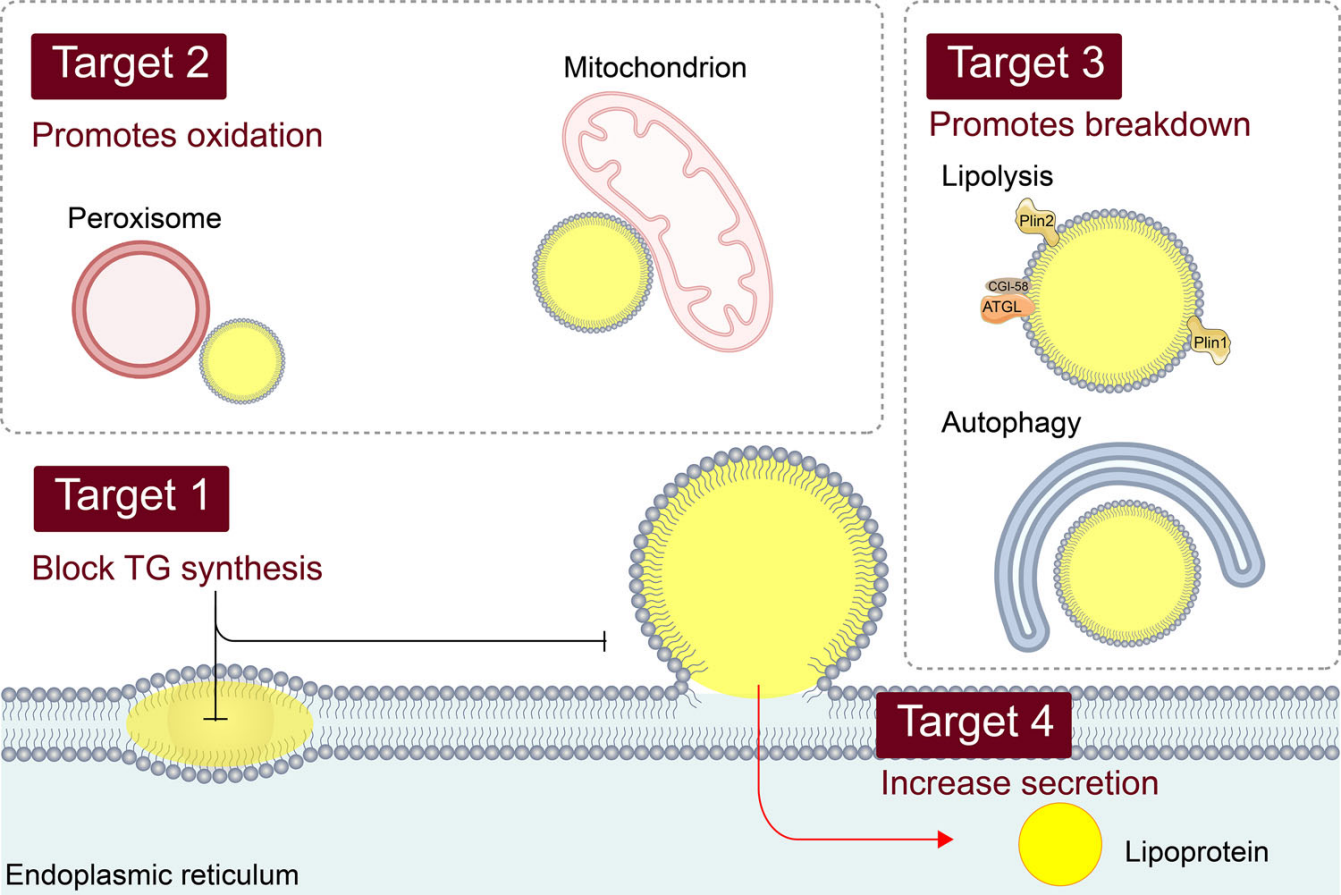
Lipid droplets (LDs) and potential therapeutic targets for metabolic diseases. Excessive deposition of lipids is a common feature of many diseases. Appropriate intervention in the three processes of LD formation, growth and catabolism can provide potential measures for treatment. This includes reducing triglyceride synthesis, promoting lipoprotein secretion, activating fatty acid oxidation with mitochondria and peroxidase and promoting lipolysis and autophagy.

LDs are widely implicated in metabolic‐related diseases, particularly obesity, type II diabetes, atherosclerosis, cardiovascular disease, kidney diseases and NAFLD, as they serve as sensors of cellular nutrient status.^33^ Excess lipid storage results in the accumulation of LDs, which can cause inflammation. In addition, LDs have been identified as a source of energy for viruses and bacteria, which utilise them for replication, assembly and production of inflammatory mediators, such as Chlamydia and SARS‐CoV‐2.^34^ Notably, NAFLD is characterised by a significant accumulation of LDs, which is attributed to the over‐activated de novo lipogenesis (DNL) pathway mediated by sterol regulatory element binding transcription factor (SREBF). In general, DNL accounts for only 5% of lipids, while it is up to 26% in patients. Similarly, LDs in cancer cells are regulated by the sterol regulatory element binding protein (SREBP) and mechanistic target of rapamycin (mTOR)pathways, ultimately becoming the primary energy supply and signalling hub in these cells.^35^ The abundant lipid synthesis not only provides energy, but also generates various lipid signalling molecules, such as eicosanoids, which participate in immune and inflammatory processes.^36^ The growing body of evidence indicates that LDs not only play a role in cancer metabolism but also may become potential targets for cancer therapy in the future.^37^ Each step of the LD life cycle is related to disease progression, which includes its formation, growth and breakdown. The molecular mechanisms of these processes can provide additional opportunities for therapeutic intervention in related diseases (Figure 3).

## 4 VISUALISATION AND FLUORESCENT PROBES OF LIPID DROPLETS

Reliable tools for investigating the functions of LDs are necessary to provide researchers with a more comprehensive understanding of these organelles. Visualising LDs is a commonly used approach that allows for direct observation of cellular LDs under various types of microscopy, including conventional light microscopy, phase contrast microscopy, Raman microscopy and electron microscopy.^38^ While these techniques provide direct observation of intracellular LDs, they do not allow for localisation and interaction analysis with other organelles or proteins. Immunofluorescence staining is a widely used biochemical method for laboratory detection of target molecules. The presence of PLIN family proteins on LDs enables researchers to localise LDs via immunofluorescence, particularly using the Plin2 protein, which is specifically distributed on LDs and is often employed as a surface marker for LDs. This method permits the labelling of multiple organelles simultaneously, such as the ER, mitochondria and nucleus. However, common immunofluorescence protocols require cell immobilisation, which may affect LD morphology. Furthermore, the permeabilisation step may result in the non‐detection of a small fraction of proteins. Additionally, this method is not suitable for live cell studies and dynamic detection of LDs.^39^

**FIGURE 4:**
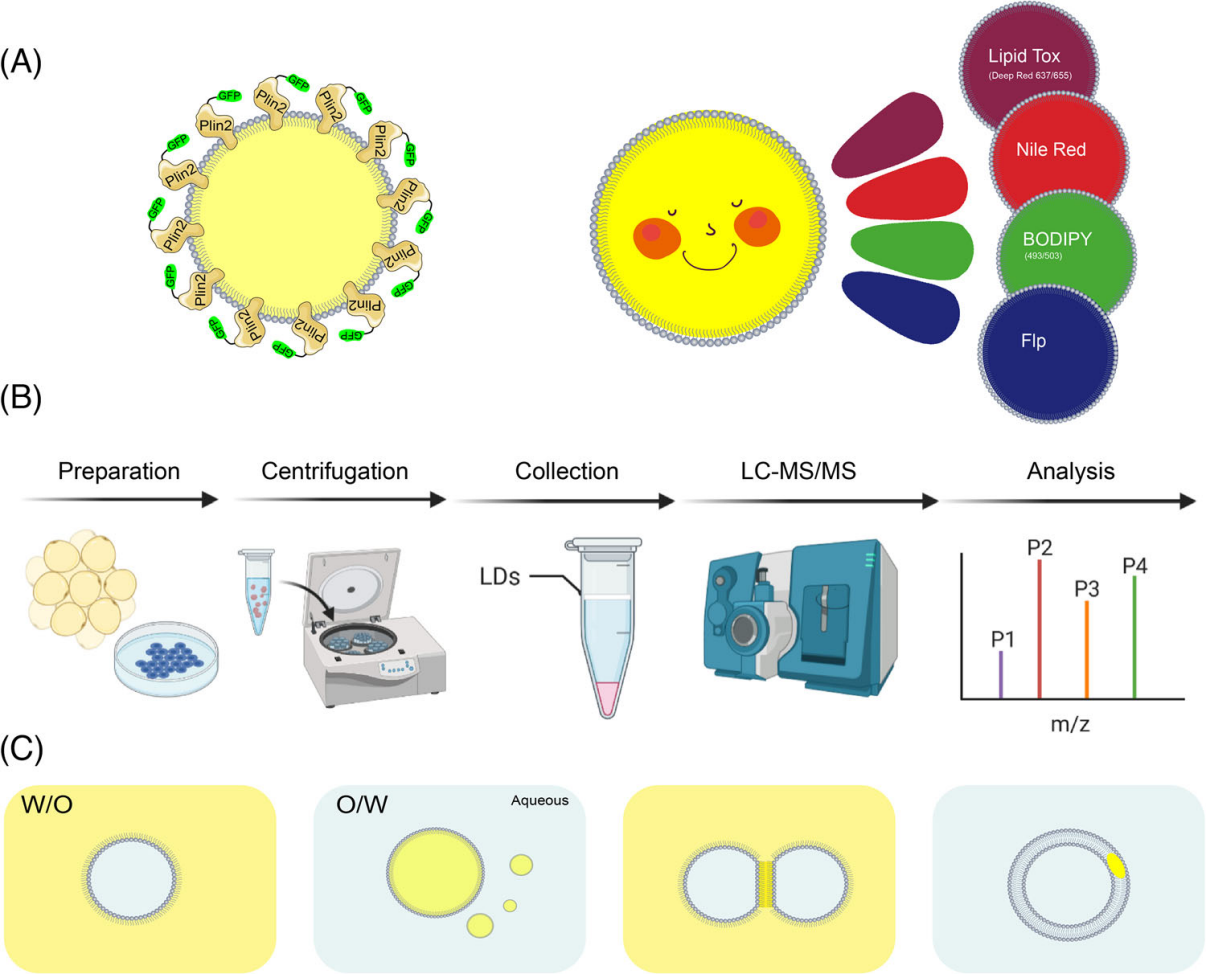
Common methods for investigating lipid droplets (LDs). (A) Visualisation of LDs. Visualisation of LDs is generally performed by two methods. One is immunofluorescence by labelling the LD‐specific protein Plin2. Or by fluorescent dyes such as Nile Red, BODIPY and LipidTox. (B) The separation and purification of LDs requires three main steps: first, sample preparation and fragmentation; second, separation of LDs and other cellular fractions by ultracentrifugation; and finally, collection and purification followed by subsequent experiments. (C) There are two mainstream models for in vitro construction of LDs, oil‐in‐water (O/W) and water‐in‐oil (W/O). Based on these two models, there are new bilayer models for droplet interface bilayer and droplet‐embedded vesicles (DEVs).

The investigation of LDs has been greatly facilitated by the development of chemical tools, particularly fluorescent probes, which enable researchers to visualise and track LDs with ease.^40^ Traditional dyes such as Sudan III and Oil Red O^41^ have been used to stain LDs, but they have been found to disrupt the LD structure during staining.^42^ Nile Red is also a common red probe that fluoresces red in most organic solvents and lipid environments.^43^ However, its susceptibility to non‐specific staining of other lipid‐containing organelles renders it unsuitable for multicolour imaging. The most frequently employed fluorescent dyes for LDs are BODIPY 493/503 and its analogue BODIPY 505/515. These dyes exhibit a bright green fluorescence and have been shown to label LDs rapidly and reliably, although they are susceptible to generating background signals. Recently, LipidTox NLs stain was developed by Thermofisher, which can label LDs at different wavelengths (green: λex/em = 495/505, red: λex/em = 577/609 and dark red: λex/em = 637/655), but its structure has not been disclosed. LipidTox is more specific for LDs, and its far‐infrared band can be advantageous in saving valuable red or green channels for labelling additional proteins or organelles. However, its limitation is that it can only be utilised to stain fixed cells, and the fluorescence tends to quench (Figure 4).^44^

The labelling of LD contact and interaction with other organelles helps us to understand the function of LDs. However, normal fluorescence imaging is limited in the number of labels to distinguish in a single image, so the spatial and temporal organisation characteristics of LDs remain incomplete. The development of new imaging devices and analysis software has helped to address this challenge. Researchers have achieved the mapping of LD number/volume/velocity/position and dynamic inter‐organelle contacts (ER, mitochondria, LDs, Golgi apparatus and lysosomes) in living fibroblasts by confocal and lattice light sheet instrumentation and an imaging informatics pipeline of five steps.^45^ Visualisation of multiple organelles contributes to the characterisation of the cellular distribution of LDs under different conditions, and guides investigators in targeted molecular mechanism studies.

## 5 ISOLATION AND PROTEOMIC ANALYSIS

A new frontier in the study of LDs has been opened up by the isolation of LDs from cells or tissues and subsequent proteomic analysis.^46^ However, this research area presents two important challenges and difficulties. The first challenge is obtaining pure LD protein fractions, which is crucial for effective LD separation methods that reduce contamination by spurious proteins and provide pure samples for mass spectrometry.^47^ LD isolation methods for different cell and tissue types have been described and commonly involve density gradient centrifugation, as LDs have a lower density than other cellular fractions.^48^ Nonetheless, this method inevitably leads to contamination with other non‐LD proteins. To overcome this, investigators have utilised APEX2 to label LD proteins and identified a high‐confidence LDs proteome through proteomic association analysis, thereby avoiding common contaminating proteins. These methods have identified over 150 LD proteins.^49^ The second challenge is mapping the proteins of heterogeneous LD subpopulations. LDs vary in size, localisation, status and organelle contacts even within the same cell, and this heterogeneity is lost during LD isolation. However, LDs within the same cell have been separated into three fractions based on size and demonstrated their function.^50^

## 6 TWO MODELS OF SYNTHETIC LIPID DROPLETS

### 6.1 Artificial lipid droplets with monolayer phospholipid

**FIGURE 5:**
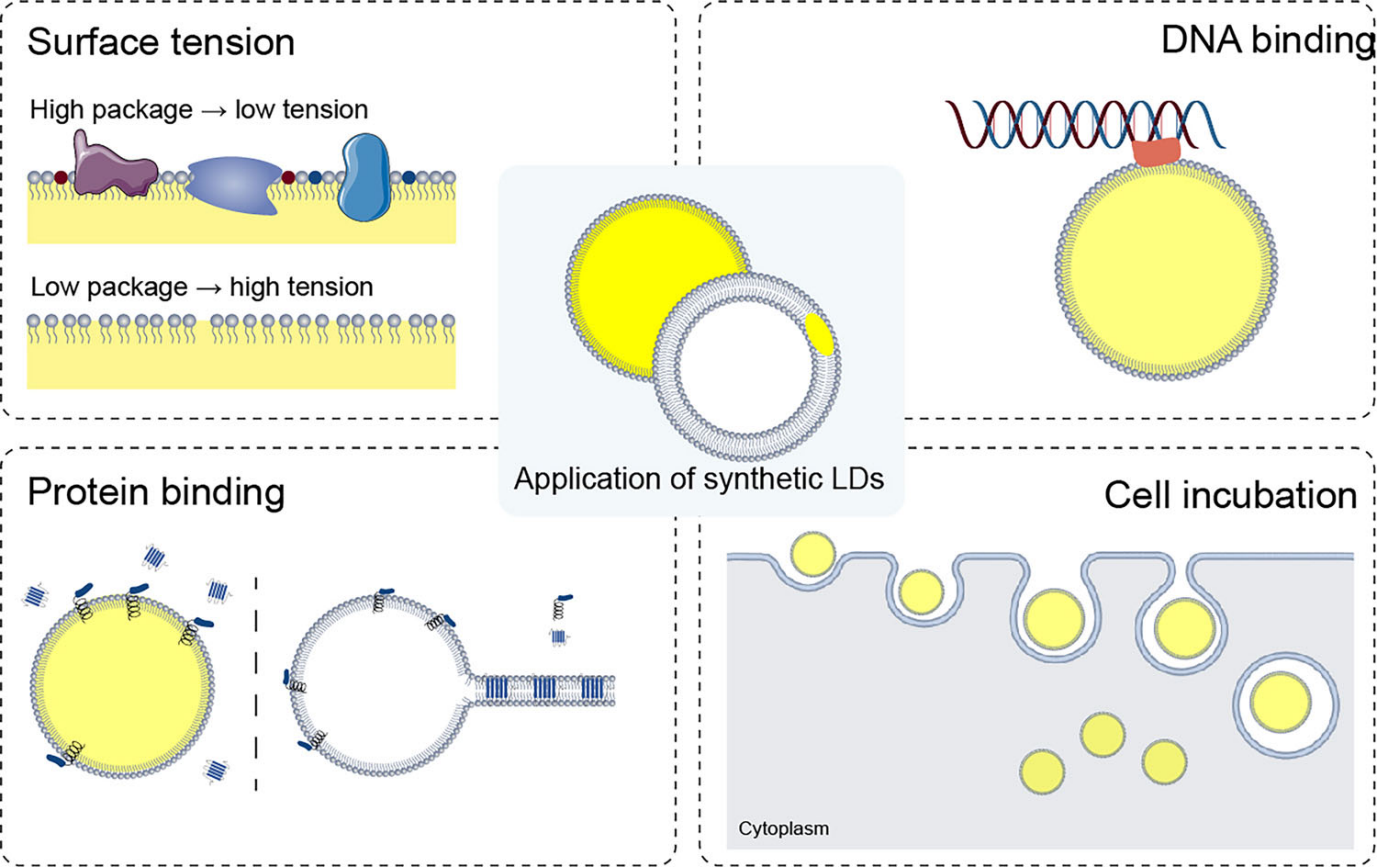
Model of in vitro lipid droplet (LD) construction. There are usually two models for the in vitro reconstruction of LDs: aLDs containing a monolayer of phospholipid (PL) membranes and droplet‐embedded vesicles (DEVs). aLDs are excellent mimics of endogenous LDs because of their high similarity in structure and lipid composition. It has been widely utilised in LD biology, especially protein interactions, DNA binding and incubation of cells as biocompatible carrier. The DEV model demonstrates the distribution and assembly of lipids in bilayers, which is consistent with the generation of LDs from the endoplasmic reticulum (ER). Using the DEV model, the researchers have explored the influences on LD budding from a biophysical perspective and have achieved excellent results, confirming the way in which lipid and protein fractions affect LD budding through surface tension.

Several researchers have successfully constructed emulsification systems that encapsulate LD proteins to form artificial LDs, using PLs as emulsifiers and either TAG or water dispersed in the medium. The predominant approach has been to create oil‐in‐water (O/W) emulsions, where the TAG is emulsified and dispersed in an aqueous phase (Figure 5). This method is commonly used to investigate the function of LDs and their surface proteins. In 1992, researchers first generated LD‐like structures in vitro by extracting plant TAG, PLs and plant protein oleosins, which they named oil bodies, and then performed studies on plant seed lipids.^51^ In 2003, a cell‐free system based on microsomes was developed to generate LDs in vitro and identify the functions of caveolin 1/2, vimentin and phospholipase D in LD formation. The role of PA in controlling LD size was later discovered and demonstrated using artificial lipid emulsions.^52^ In 2011, a similar lipid emulsion was used to study the role of CCTα, and it was found that PC can act as a surfactant to stabilise and prevent LD agglutination.^53^ Researchers have also used microfluidic devices to generate O/W LDs and identified that Arf1 binds to the TAG/buffer interface in a guanosine triphosphate (GTP) ‐dependent manner, and that Cy3‐labelled Arf1 cannot bind to LDs when GTP is absent from the medium. Similarly, the lipid‐binding structural domain (HDS1) of the Arf1 regulator GBF1 was identified in vitro to bind to the monolayer PL surface, which mediated the binding of LDs to the Golgi membrane.^54^ In 2016, an in vitro reconstitution method for LDs, called adiposomes, was established, which can rapidly form highly stable artificial LDs for downstream experiments. Using adiposomes,^55^ researchers investigated the role of microorganism LD small protein (MLDS) in the recruitment of DNA. The authors co‐incubated artificial LDs with DNA and purified MLDS proteins, collected the droplets by centrifugation, and found that LD‐recruited DNA was MLDS dependent. The preparation of artificial LDs not only elucidated the function of the protein but also solved the problem that bacterial LDs are too small to be observed.^56^ Additionally, the function of the ABHD4 N303R/S332G mutation in stimulating lipolysis was verified using an in vitro system, which was found to stimulate ATGL‐dependent lipolysis by detecting free fatty acid (FFA) in the system, albeit with weaker intensity than the purified ABHD5.^57^ Recent studies prepared LDs using a microfluidic device and used them as materials to activate B cells and verified the effects of different physical and chemical properties on lysosomal polarisation of B cells.^58^

The refinement of in vitro methods for preparing LDs has been an ongoing process aimed at tailoring these methods to diverse experimental requirements. Incorporation of SE and cholesterol into artificial LDs poses a challenge due to their high melting point and low solubility. To address this issue, the original method has been modified, leading to the successful generation of stable LDs containing SE and cholesterol via reversed‐phase evaporation and sonication.^59^ Additionally, by increasing the amount of oleosin purification and adjusting the molar ratio of oil to PL, the synthesis of oil bodies (LDs in plants) that are less prone to aggregation has been achieved.^60^ The conventional thin‐film dispersion method, which dissolves lipid with chloroform and then forms a thin film through nitrogen gas drying, followed by ultrasonic shaking with an aqueous buffer to create artificial LDs, generates a significant amount of contaminants and necessitates multiple centrifugation steps to obtain pure LDs. However, a novel LD preparation technique based on the solvent injection method has been developed. This approach produces highly stable artificial LDs with uniform particle sizes and facilitates easy isolation of pure LDs without impurities.^61^ In some studies, a water‐in‐oil (W/O) model has also been employed. Here, W/O droplets were generated using 1,2‐Di(9z‐Octadecenoyl)‐Sn‐Glycero‐3‐Phosphoethanolamine (DOPE), 1,2‐Di(9z‐Octadecenoyl)‐Sn‐Glycero‐3‐Phosphocholine (DOPC) and mineral oil, and then co‐incubated with purified monomers of desmin and actin. In the absence of metal ions, desmin was uniformly distributed in the droplets. However, upon the addition of metal ions, desmin rapidly assembled into clusters located at the inner edge of the droplet within 2 min. Furthermore, protrusions from the region of desmin accumulation to the outer space were observed, indicating that the accumulation of desmin at the periphery is crucial for altering LD shape.^62^

In addition to protein identification, artificial LDs offer a valuable tool for functional study of LDs. The highly dynamic and intricate membrane system of the cell means that changes in lipid composition often reflect the cellular state, making it challenging to draw firm conclusions on LDs from intracellular experiments. Artificial LDs resolve this issue by demonstrating that different lipid compositions impact LD size and lipid peroxidation, with unsaturated lipids leading to more intense peroxidation. This study offers compelling evidence linking LD lipid composition with susceptibility to oxidation.^63^ Furthermore, the role of PI (PtdIns) in LD function has been elucidated, revealing that an increase in PI content reduces Plin2 binding to LDs, while having no effect on its cognate family protein, Plin3. This is due to the fully exposed residue E73 of Plin2, which affects binding to PI‐containing membranes, unlike Plin3. Additionally, ATGL exhibits greater lipolytic activity towards PI, which is closely linked to its S47 enzyme active site and S87 phosphorylation site.^64^ Moreover, incubating cells with artificial LDs and measuring reactive oxygen species (ROS) levels revealed that LDs themselves possess ROS resistance, consistent with their role in regulating cellular stress. In vitro experiments demonstrated that this effect is non‐protein dependent.^61^

### 6.2 Droplet‐embedded vesicle model

Incompatibility between oil and water is a well‐established physical phenomenon, which necessitates a large energy investment to create an interface between them. This interfacial tension, known as surface tension, can significantly affect droplet size and stability in emulsions.^65^ In this context, lower surface tension promotes smaller droplet formation and improved stability, while higher surface tension produces the opposite effect. LDs, similar to oil–water emulsions, comprise a suspension (cytoplasm) and oil (NLs) stabilised by a surfactant (PL monolayer).^66^

To minimise energy expenditure, the surface tension limits contact area at the interface, thereby ensuring the spherical shape of LDs.^67^ While it remains unclear whether the formation and stability of LDs in vivo are governed by physical principles such as surface tension or curvature, resolving this issue represents a significant challenge for researchers. The impact of surface tension on the stability of LDs has been proposed for a considerable period, but direct evidence supporting this hypothesis has remained elusive.^68^ To address this issue, droplet‐embedded vesicles (DEVs) have been developed as an in vitro model. This approach involves embedding nano‐emulsions into PL bilayers to construct giant unilamellar vesicles that mimic cellular LDs in contact with the ER bilayer. The lipid composition and biophysical properties of DEVs can be tailored to control and adjust their vesicle interface, while the physicochemical characteristics such as curvature and surface tension can be directly measured in vitro. Furthermore, the PL monolayer in the droplet inlay and the surrounding bilayer region exhibit distinct physicochemical properties, enabling direct investigations into protein targeting.^69^ Using the DEV model, researchers have provided compelling experimental evidence for the impact of surface tension on the nucleation and growth of LDs. By employing various lipid components to create DEVs with varying surface tensions, the investigators observed significant changes in the shape of the lipids inserted between the bilayers. Specifically, the data showed that low surface tension promoted LD germination. To corroborate these findings, the researchers added exogenous phospholipase A2, which facilitated the transition from PC to lyso‐PC to reduce surface tension. As anticipated, this manipulation led to an increase in LD growth, as confirmed by subsequent intracellular experiments.^70^ The DEV model links the physicochemical property of surface tension to the formation of LDs and enables direct measurement of the surface tension of artificial LDs in vitro, thereby providing a means to validate scientific hypotheses by modifying the in vitro system.

In addition to lipid composition, various factors, such as membrane packaging, protein attachment and curvature can also influence membrane surface tension. In vivo, LDs tend to bud towards the cytoplasmic side of the ER membrane, which suggests that there exists a surface tension difference between the two sides of the ER membrane at the budging site. This observation is corroborated by the DEV model, which has demonstrated that LDs preferentially bud from the side with lower surface tension. Specifically, researchers have found that LDs always bud from the side with higher PL surface density and more adequate protein attachment, which suggests that these factors contribute to lower surface tension. Maintaining proper asymmetric surface tension is critical for preserving the ER's normal morphology and facilitating proper LD budding.^71^ These results align with earlier intracellular studies on seipin, which indicates that seipin forms a scaffold around nascent LDs to maintain their morphology. Overall, the DEV model has provided important experimental evidence for the role of surface tension in LD formation and supports the idea that asymmetric surface tension plays a key role in this process.

Recent studies have shown that most nascent LDs are located in the tubular ER, and even when they present in the sheet, they are found at the highly curved edges.^72^ The degree of curvature of the membrane is a key factor that influences LD formation, with larger curvatures leading to smaller vesicles. Studies on LD biogenesis have revealed that almost all nascent LDs originate from small tubular ER structures, and only a few are formed in larger vesicles, suggesting that membrane curvature may impact affect NL nucleation. Despite this compelling evidence, confirming this hypothesis remains a challenge. The DEV model represents a valuable tool for studying the kinetics of LD budding as well as for tackling the issue of LD protein targeting. By adding tubular structures to the DEV model, researchers have observed a decrease in TAG motility in tubules compared to smooth interfaces, indicating that high membrane curvature promotes TAG nucleation and LDs budding.^73^ Moreover, the use of the DEV model has led to new insights into the selective targeting of proteins to LDs. Proteins binding to LDs through amphipathic helix (AH) structures engage in a selective targeting process since the NLs of LDs may be temporarily exposed to the surface, causing an increase in surface tension associated with PL packaging defects. Hydrophobic regions of AHs anchor in voids to diminish surface tension, and hydrophobic residues are essential for targeting of AHs.^74^ The preference of AHs for NLs determines their binding level to LDs, thereby enabling LDs to regulate protein binding by modulating the packing level of PLs. This mechanism not only elucidates the specific process of protein targeting to LDs, but also prevents excessive protein binding, which is hindered by macromolecular crowding on the LD surface.^75^, ^76^ Similar to DEVs, lipid sponge droplets can mimic the membrane structure in the ER with a dense meshwork of lipid bilayers and nano‐water channels. It can contain functional soluble and transmembrane proteins that allow co‐localisation and concentration of enzymes and substrates to enhance reaction rates. This structural feature provides an excellent mimicry of the environment of LDs in the cell, especially contact with the ER and enzymatic reactions.^77^ Although relevant studies on LDs have not been reported, these findings are of great significance for enhancing our understanding of LD function and assembly.^78^

## 7 APPLICATION OF SYNTHETIC LIPID DROPLETS

The aforementioned investigations only scratch the surface of LDs' functionality, yet they aptly demonstrate the impact of protein and lipid composition on membrane curvature, asymmetry and protein binding, which allows us to establish a connection between LDs' entire life cycle and lipid fluctuations stemming from dietary or pathological conditions. Ascertaining how these factors are modulated by changes in nutrients, ER stress, diseases or genetics is pivotal, and further exploration of the signalling pathways and molecules involved in these factors represents a crucial avenue of future research. Development and implementation of drugs targeting these pathways may pave the way for treating a wide range of disorders, including obesity, metabolic diseases, NAFLD and cancer.

LDs have been identified as essential cellular structures capable of serving as reservoirs for intracellular hydrophobic drugs. Lasonolide A (LasA), a macrolide antibiotic with potent antitumour activity, is one such drug that can accumulate in LDs. The hydrolysis of LasA in cells is mediated by LD‐associated hydrolase (LDAH), which is located on the surface of LDs. Upon entry into cells, LasA is preferentially enriched in LDs, and LDAH converts it into the active cleavage product LasF, which mainly acts in the cytoplasm. This selective enrichment of hydrophobic drugs in LDs indicates their importance as phase separation compartments and highlights their key biochemical functions.^79^ Notably, the anti‐tuberculosis drug Bedaquiline accumulates mainly in LDs of infected human macrophages, where it acts as a storehouse and enhances the cells' antibacterial effect.^80^ These studies indicate that LDs can act as drug ‘storage’. Targeting LDs could be used to achieve slow release and activation of drugs in cells.

It is predictable that LDs can be used as drug delivery devices. Researchers have developed a bionic AIE photosensitiser DC@AIEdot with antigen presentation and ‘hitchhiking’ functions by encapsulating dendritic cell (DC) membranes on nanoclusters of aggregation‐induced luminescent molecules (AIEgens). This novel approach results in a 1.6‐fold increase in delivery efficiency, stimulating T‐cell proliferation and activation in vivo, and triggering an immune response. This study highlights the potential of LD‐targeted therapeutic agents in immunotherapy and presents a new approach for tumour treatment.^81^ Moreover, extracted LDs have been demonstrated to act as controlled and biocompatible carriers for the delivery of anticancer drugs, improving the efficacy of cancer photodynamic therapy.^82^

The generation of LDs alleviates cellular lipotoxic stress by promoting protein turnover as well as protecting polyunsaturated fatty acids (PUFAs) from damage caused by ROS.^83^ ROS attack the double bonds in PUFAs, leading to lipid peroxidation, which disrupts normal cellular function and leads to cell death under certain conditions. LDs are less susceptible to peroxidation than cell membranes, so phospholipases can release PUFAs from cell membranes to be stored in LDs for protective effects.^26^ Synthetic LDs can be used as anti‐stress agents. Cells incubated with aLDs significantly reduced ROS induced by H_2_O_2_ and alleviated lipotoxicity induced by excess FA stimulation.^61^

Another undesirable consequence of excess ROS is the resulting damage to membrane lipids. Normally, intracellular antioxidants neutralise ROS. However, under pathological conditions, the failure of this regulatory effect causes membrane damage. Therefore, it must be repaired to maintain membrane structure and function. Synthetic LDs could perhaps be developed into an oral dietary supplement that would provide enough membrane glycerophospholipids to repair damaged lipids and return cell membranes to normal function.^84^ The high biocompatibility of synthetic LDs promotes drug uptake by cells and tissues. It can also be used as an assistant carrier for traditional drugs to increase the targeting of drugs to cells and make them more effective. What's more, this organelle‐derived mimic is so safe that there is no need to consider its safety.^85^ Recent studies have demonstrated the use of synthetic polymers to directly isolate proteins and lipids from the cell membrane.^86^, ^87^ Such nanoparticles, which are called nanodiscs, are excellent membrane mimetics and are used for various applications. The application of this technique to synthetic LDs would allow researchers to more precisely mimic subcellular compartments and better resolve the dynamic function of LDs in the cell.^88^

## 8 CONCLUSIONS AND FUTURE DIRECTIONS

**FIGURE 6:**
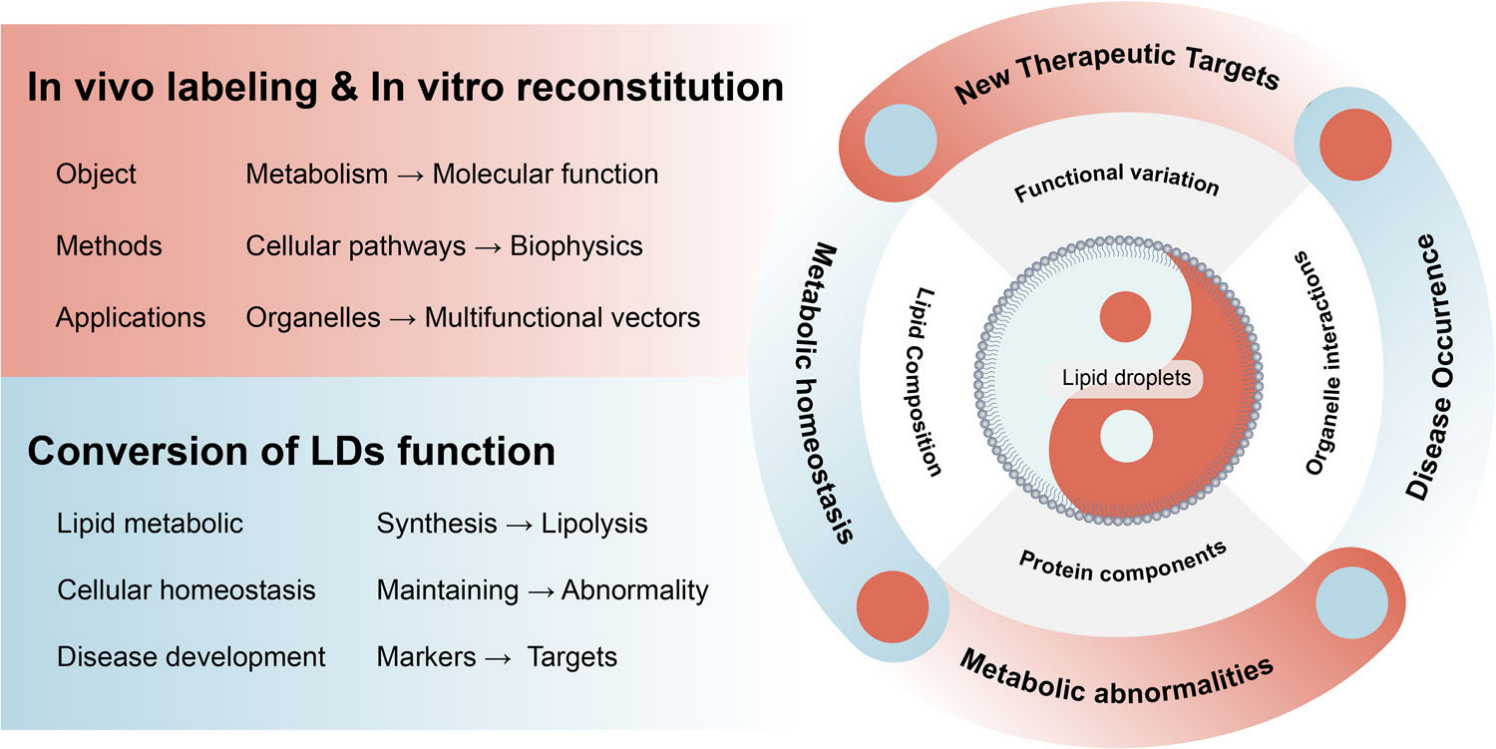
Lipid droplets (LDs) as a new breakthrough in metabolic research. LDs are a core organelle of lipid metabolism that can be investigated by both in vivo labelling and in vitro reconstitution. Its function changes dynamically according to the nutritional status of the cell, and under normal conditions it plays a role in storing lipids, maintaining energy homeostasis, and alleviating cellular stress. However, under pathological conditions, excessive deposition of LDs causes metabolic disturbances and disease development. In vitro remodelling of LDs has made significant progress in protein binding, lipid function, membrane dynamics and enzymatic reactions, and is becoming a new breakthrough in metabolic research.

Recent years have witnessed significant advancements in the study of LDs, particularly regarding the mechanisms of biogenesis and organelle interactions. The existing consensus is that alterations in nutritional status significantly influence the life cycle of LDs, both in their lipid and protein fractions (Figure 6). Despite this consensus, the underlying mechanisms remain elusive. Novel experimental techniques have generated promising outcomes in vitro. It is anticipated that in vitro models of LDs will be further refined in the future and that an increasing number of researchers will be drawn to investigate the intriguing aspects of LDs. However, several questions still require further elucidation, such as how do cells evaluate the curvature and size of LDs? How does LD heterogeneity relate to cellular state? What are the mechanisms underlying the emergence of heterogeneous lipid and protein fractions, and how do they affect LD function? Clarifying these and other questions yet to be posed may facilitate a comprehensive understanding of the role of LDs in diverse events, notably metabolic disorders, cardiovascular disease, cancer and neurodegeneration, and may lead to the development of strategies for preventing and combating these debilitating diseases.

## AUTHOR CONTRIBUTIONS

Conceptualization: PX Z and ZQ R; Writing—original draft: PX Z, ZZ C and ZW Y; Writing—review & editing: PX Z and ZQ R; Funding acquisition: ZQ R; Project administration: Y J, J W and ZQ R; Visualization: PX Z and LP C.

## CONFLICT OF INTEREST STATEMENT

The authors declare they have no conflicts of interest.

## Data Availability

Not applicable.
